# The remineralization effect of GERM CLEAN on early human enamel caries lesions in vitro

**DOI:** 10.1038/s41598-023-31405-1

**Published:** 2023-03-13

**Authors:** Ye Wang, Kaixin Xiong, Xuan Chen, Yaqi Chi, Qi Han, Ling Zou

**Affiliations:** 1 State Key Laboratory of Oral Diseases & National Clinical Research Center for Oral Diseases, Department of Conservative Dentistry and Endodontics, West China Hospital of Stomatology, Sichuan University, Chengdu, 610041 China; 2 State Key Laboratory of Oral Diseases & National Clinical Research Center for Oral Diseases, Department of Oral Pathology, West China Hospital of Stomatology, Sichuan University, Chengdu, China

**Keywords:** Dental caries, Dental biomaterials, Enamel

## Abstract

This study aimed to evaluate the remineralization effect of GERM CLEAN, a novel antibacterial peptide, on early enamel caries. Thirty human enamel blocks from thirty teeth were randomly divided into three groups: double distilled water (DDW group), GERM CLEAN (GC group), and 1000 ppm fluoride (NaF group). Specimens were demineralized for 3 days (pH 4.6) followed by pH cycling twice daily for 14 days. For a pH cycle, specimens received corresponding treatments for 5 min, then were immersed in demineralizing solution for 1 h, received corresponding treatments again, and finally were immersed in remineralizing solution (pH 7.0) for approximately 11 h. Specimens were washed with DDW after each treatment. Microindentation tests, atomic force microscopy (AFM), and transverse micro-radiography (TMR) were conducted to analyze enamel blocks. GC demonstrated a lower percentage of surface microhardness recovery (SMHR%) (p < 0.0001), rougher surfaces (p < 0.0001), deeper lesion depth (p = 0.001), and more mineral loss (p = 0.001) than NaF, but showed higher SMHR% (p < 0.0001), smoother surfaces (p < 0.0001), shallower lesion depth (p = 0.049), and less mineral loss (p = 0.001) than DDW. As a result, GERM CLEAN has the potential to promote the remineralization of demineralized enamel.

## Introduction

Dental caries is a chronic progressive destructive disease that occurs in dental hard tissue under the influence of multiple factors dominated by bacteria. In the early stage of dental caries, acid produced by bacteria breaks the dynamic balance between enamel demineralization and remineralization, resulting in enamel demineralization at the subsurface^1^. Early enamel caries is clinically characterized by the white spot lesion^2^, which not only affects appearance but also affects dental health. Due to the non-renewable characteristics, enamel cannot restore the physical, chemical, and mechanical properties spontaneously^3^. At present, for the treatment of white spot lesions, the most commonly used methods in clinical practice include plaque control, diet management, and topical fluorides^4^.

Fluoride is currently the most widely used clinical strategy to counteract early enamel caries^5^. According to the previous study, fluoride can not only inhibit enamel demineralization but also enhance enamel remineralization^5^. Fluoride will adsorb to the surface of the partially demineralized crystals and attract calcium ions to form a calcium fluoride-like layer on the enamel surface^5,6^. But not all population groups are suitable for fluoride such as people who are allergic to fluoride^7^. Other reported management measures for white spot lesion like dental bleaching^4^, microabrasion^8^, resin infiltration^8^, and direct or indirect restorations^4^ have some disadvantages. Vital tooth bleaching has the risk of increasing bleaching sensitivity^9^. Microabrasion is an effective method for superficial lesions and should not be used where the enamel is thin^10^. Resin infiltration seems to be feasible for early enamel lesions^11–14^; however, the infiltrant could not form a smooth coating on the lesion’s surface even after being polished with finishing trips^15^. It was reported that resin infiltration could not restore the surface hardness of demineralized enamel to that of sound enamel^16^. The possible reason is that the formation of polymeric chains does not always happen in the entire lesion^17^, and the polymerization shrinkage of materials during the curing process is another problem^16^. Direct and indirect restorations are the most destructive methods with the greatest tooth tissue loss compared with other options mentioned.

Due to the limitations described above, rising attention has been paid to the biomimetic mineralization of enamel, which is aimed to repair demineralized enamel by inducing the remineralization of hydroxyapatite on the tooth surface. For example, amelogenin could promote the orientated nucleation of calcium phosphate on the enamel surface^18^. The amelogenin-derived peptide, QP5, could temporarily stabilize the formation of amorphous calcium phosphate and eventually converted it into hydroxyapatite crystals^19^. Kind et al. designed a self-assembled peptide to form three-dimensional scaffolds at the subsurface lesion of dental enamel, leading to the nucleation of hydroxyapatite^20^. In addition, hydroxyapatite-anchored dendrimer could adsorb on the enamel surface to form the in situ hydroxyapatite regeneration sites for further biomineralization^21^. However, the preparation of protein/peptide is difficult, and these materials are still under pre-clinical study.

GERM CLEAN (Scisyn, Chengdu, China) is a novel oral spray, the active component of which is a new synthetic polypeptide. According to the manufacturer’s instructions, this product can effectively kill a wide range of pathogenic bacteria that triggers oral mucositis and periodontitis. Recent studies have shown that GERM CLEAN has an obvious antibacterial effect on *Streptococcus mutans*^22^ and dual-species biofilm of *S. mutans* and *Candida albicans*^23^, and can inhibit the demineralization of bovine enamel^23^. However, the remineralization effect of this spray is yet to be investigated.

This study aimed to evaluate the remineralization effect of GERM CLEAN on human molar early enamel caries in vitro. Vickers microhardness, atomic force microscope, and transverse micro-radiography were conducted to assess mechanical properties, surface roughness, and mineral content, respectively. The null hypothesis was that no significant differences would be found in the remineralization effect between the double distilled water (DDW) and GERM CLEAN.

## Methods

### Specimen preparation

Thirty extracted human molars were collected from a pool of teeth with the approval of the Medical Ethics Committee of West China Stomatology Hospital of Sichuan University (WCHSIRB-D-2022-019). These teeth were stored in a 0.4% thymol solution (Macklin, Shanghai, China) before use. Informed consent was obtained from patients and the study was performed in accordance with the Declaration of Helsinki. Teeth with dental fluorosis, cracks, enamel or dentine defects, or representing any type of dental restoration or sealant were excluded.

**Figure 1:**
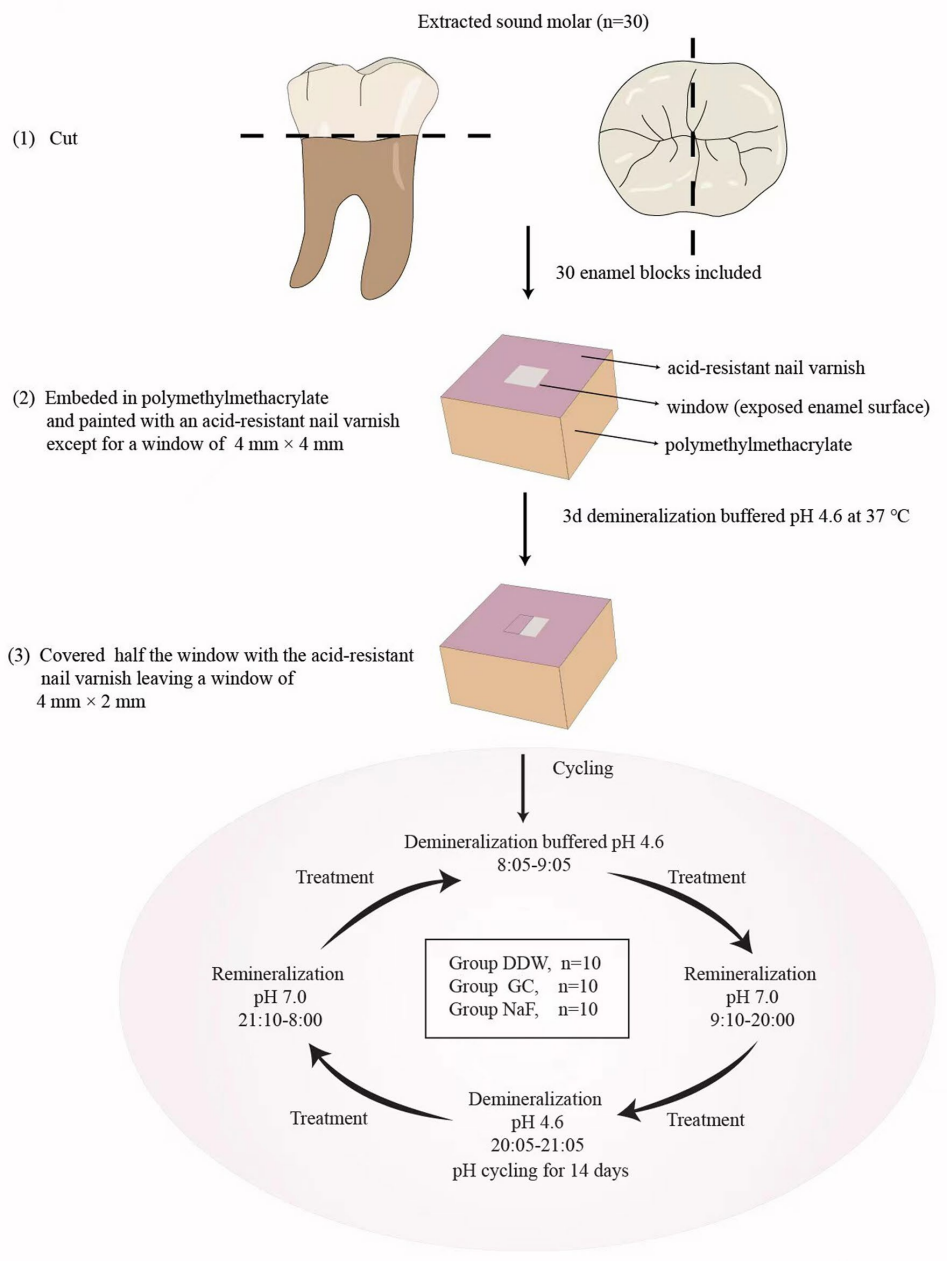
The flowchart of the experimental procedure.

The teeth were de-coronated and each crown was cut into two sections by a low-speed cutting machine (Struers Minitom; Struers, Copenhagen, Denmark) under running deionized water to generate sixty enamel blocks. Eight enamel blocks that were too small or with defects and cracks were discarded and fifty-two enamel blocks were produced. These enamel blocks were embedded in polymethylmethacrylate (Macklin, Shanghai, China), followed by grinding with water-cooled carborundum discs (Struers Minitom; Struers, Copenhagen, Denmark) of 1200 grit waterproof silicon carbide paper (Yu Ying, Foshan, China) to form a window of 4 mm × 4 mm on the enamel surfaces (Fig. 1). The entire enamel surface but the window was then painted with two layers of acid-resistant nail varnish (MINISO, Tokyo, Japan).

Before artificial enamel caries formation, the baseline surface microhardness (SMH_0_) of the enamel blocks was measured. Three indentations spaced 300 μm from one another were made at the center of the window. Measurements were taken with a Vickers micro-hardness tester (Matsuzawa, Akita-ken, Japan) at a load of 50 g for 15 s. Finally, thirty enamel blocks with hardness ranging between 360 to 390 VHN were selected for further study.

### Artificial enamel caries formation

The early enamel caries lesions were produced by the method described by Lv et al.^24^. The selected enamel blocks were immersed in demineralizing solution (2.2 mM KH_2_PO_4_, 2.2 mM Ca(NO_3_)_2,_ 50 mM acetic acid, pH 4.6) at 37 °C for three days to produce artificial enamel caries under continuous, low-speed magnetic stirring (100 rpm) (ZHICHENG, Shanghai, China). The surface microhardness of the enamel blocks after demineralization (SMH_1_) was measured according to the parameters mentioned before. Half the area of the exposed window on the enamel surface was painted with 2 layers of acid-resistant nail varnish (MINISO, Tokyo, Japan), leaving an enamel-exposed window of only 4 mm × 2 mm.

### pH cycling

The pH cycling procedure was consistent with the method used by Lv et al.^24^. These enamel blocks were randomly divided into three groups (n = 10) and were treated with different experimental solutions: (1) DDW (pH 6.2), (2) GERM CLEAN (GC), (3) 1000 ppm NaF (NaF) (pH 5.5). The solutions were applied topically with a volume of 40 μl to cover the aforementioned 4 × 2 mm^2^ windows in the following experimental procedures. Firstly, the three groups received treatment with the corresponding solutions for 5 min. Secondly, these specimens were immersed in demineralizing solution at 37 °C for 1 h with continuous, low-speed magnetic stirring (100 rpm) (ZHICHENG, Shanghai, China), after which, the specimens received treatments of the corresponding solutions for 5 min again. Finally, the specimens were immersed in remineralizing solution (20 mM HEPES, 0.9 mM KH_2_PO_4_, 1.5 mM CaCl_2_, 130 mM KCl, pH 7.0) at 37 °C for approximately 11 h with continuous, low-speed magnetic stirring (100 rpm) (ZHICHENG, Shanghai, China) to complete a pH cycle. The pH cycle was repeated twice daily for 14 days. Specimens were washed with DDW after each treatment. Demineralizing and remineralizing solutions were refreshed each pH cycle and 1000 ppm NaF was prepared the first time it was used.

### Surface microhardness (SMH) analysis

Surface microhardness of the enamel blocks after pH cycling (SMH_2_) was recorded under the same testing parameters and the percentage of surface microhardness recovery (SMHR%) was calculated with the formula: SMHR% = (SMH_2_ − SMH_1_)/(SMH_0_ − SMH_1_) × 100%^25^.

### Atomic force microscopy (AFM) observation

AFM images of the enamel surface after 14 days of pH cycling were captured with an Atomic Force Microscopy (SPM9700; Shimadzu, Kyoto, Japan), which was equipped with an AFM silicon probe (Shimadzu, Kyoto, Japan) and a laser scanner (30 μm × 30 μm × 5 μm). The scanning size rate was 10 µm × 10 µm and 1 Hz, respectively. For each sample, five different fields were randomly selected, and the surface roughness (Ra) of each field was analyzed by the Shimadzu SPM-9700 software (Shimadzu, Kyoto, Japan). Then the average surface roughness of each sample and each group was calculated.

### Transverse microradiography (TMR) examination

The samples were cut into 150 μm-thick slices perpendicular to the exposed windows with a diamond-coated band saw (EXAKT300; EXAKT, Norderstedt, Germany), ground all the slices to a thickness of 100- to 120-μm via 2000 grit carbide-polishing papers (Yu Ying, Foshan, China), and verified using a digital micrometer (Mitutoyo, Tokyo, Japan). Each slice was fixed on Plexiglass slides (Konica Minolta, Tokyo, Japan) in a TMR sample holder (Inspektor Research Systems, Amsterdam, Netherlands). Then slices were micro-radiographed alongside an aluminum calibration stepwedge with 11 steps using a monochromatic CuK X-ray source (Philips, Eindhoven, Netherlands) operated at 20 kV and 20 mA for 30 min at a distance of 40 cm^26^. The plexiglass slides were developed for 10 min, rinsed in deionized water, fixed for 10 min in a dark room, and then rinsed in running water for 10 min followed by air drying. The X-ray films were analyzed using a transmitted light microscope with a 20 × objective (Zeiss, Oberkochen, Germany), which was equipped with a CCD camera (Canon, Tokyo, Japan). The camera was connected to a computer (TOSHIBA, Tokyo, Japan). Quantitative data of lesion depth and mineral loss were obtained by TMR Software 2006 (Inspektor Research Systems, Amsterdam, Netherlands). Three TMR traces were measured on each slice and three slices were analyzed from each enamel block.

### Statistical analysis

Data were analyzed with SPSS 26 (IBM; Armonk, NY, USA). Shapiro–Wilk and Levene tests were applied to verify the data normality and homogeneity of the variances, respectively. The SMHR%, surface roughness, and mineral loss in different groups were compared with One-Way ANOVA and LSD as the post hoc test. The lesion depth in different groups was compared with One-Way ANOVA and Dunnett’s T3 as the post hoc test. Mean value ± standard deviation was used to represent the generated data. The significance level was set at α = 0.05.

## Results

According to the outcome of Shapiro–Wilk and Levene tests, the data of the three groups met the normal distribution. Homogeneous variances were observed in the data of SMHR%, surface roughness, and mineral loss, and the variance of data of lesion depth was nonhomogeneous. The results presented were the outcome of the One-Way ANOVA test and the corresponding multi-comparison post hoc tests.

### Surface microhardness

Table 1 showed the SMHR% of the GC group was 21.6 [± 3.5], which was significantly higher than that of the DDW group of 6.0 [± 3.9] (p < 0.0001), and the NaF group showed the highest SMHR% of 39.8 [± 6.1] (p < 0.0001).

**Table 1:** The percentage of surface microhardness recovery (SMHR%) (Mean [± SD]).

Treatments	DDW	GC	NaF
SMHR%	6.0 [± 3.9]^a^	21.6 [± 3.5]^b^	39.8 [± 6.1]^c^

Different superscript letters indicate a significant difference between groups (p < 0.05). *DDW* samples treated with double distilled water, *GC* samples treated with GERM CLEAN, *NaF* samples treated with 1000 ppm fluoride.

### Atomic force microscopy

**Figure 2:**
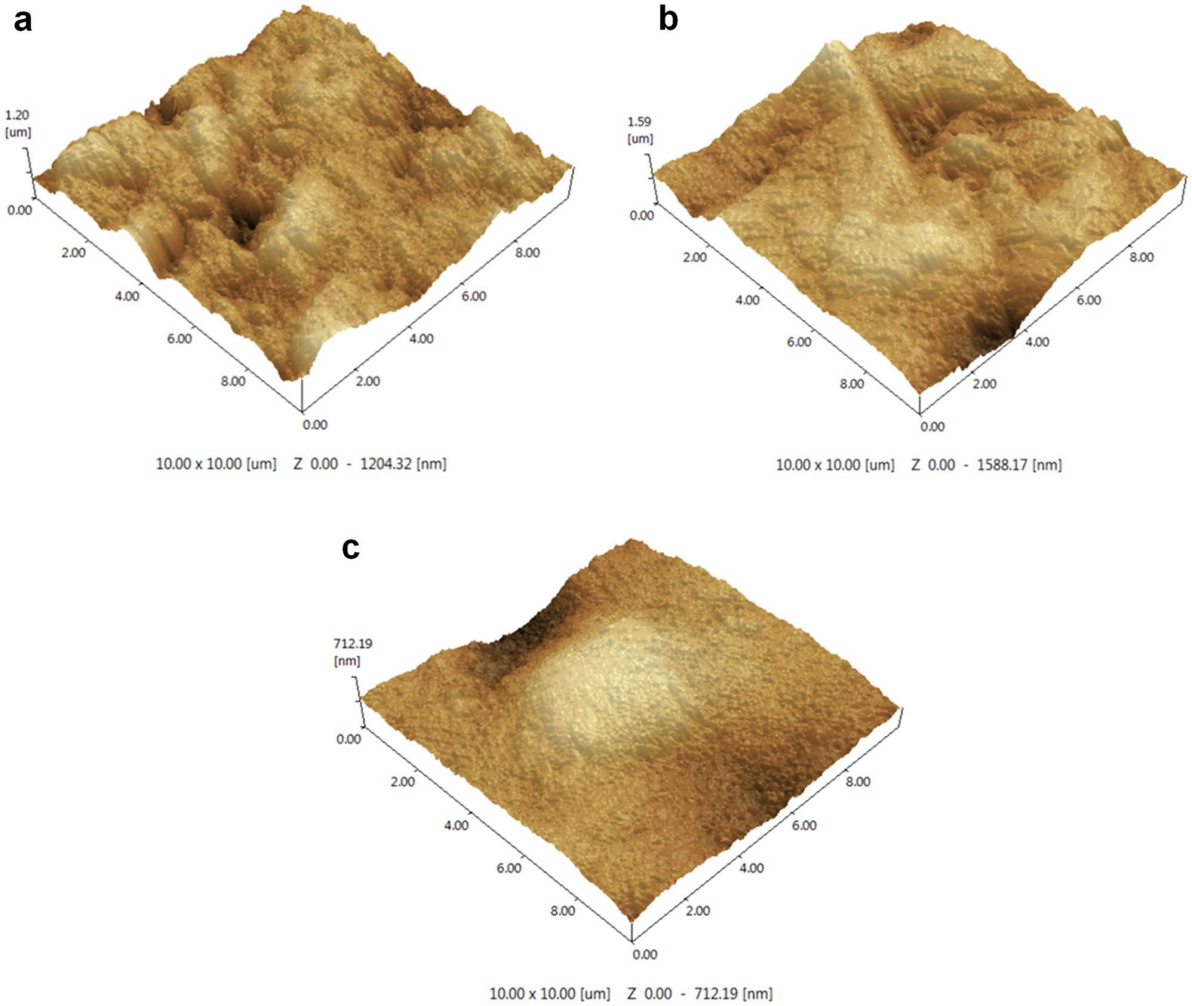
Representative AFM images (10 μm × 10 μm). The surface morphologies after 14 days pH cycling were shown in the presence of (**a**) double distilled water (DDW) alone, (**b**) GERM CLEAN, (**c**) 1000 ppm fluoride (NaF).

The surface roughness of enamel in each group was listed in Table 2. The surface roughness of the DDW group, the GC group, and the NaF group was 193.4 [± 5.4] nm, 122.3 [± 8.0] nm, and 54.5 [± 11.3] nm, respectively. As shown in Fig. 2 and Table 2, samples of the DDW group (Fig. 2a) exhibited the roughest surface, followed by samples of the GC group (p < 0.0001) (Fig. 2b), and samples of the NaF group (Fig. 2c) showed the smoothest surface (p < 0.0001).

**Table 2:** The average surface roughness (Ra) of enamel blocks after pH cycling (Mean [± SD]).

Treatments	DDW	GC	NaF
Surface roughness (Ra, nm)	193.4 [± 5.4]^a^	122.3 [± 8.0]^b^	54.5 [± 11.3]^c^

Different superscript letters indicate a significant difference between groups (p < 0.05). *DDW* samples treated with double distilled water, *GC* samples treated with GERM CLEAN, *NaF* samples treated with 1000 ppm fluoride.

### Transverse microradiography

**Figure 3:**
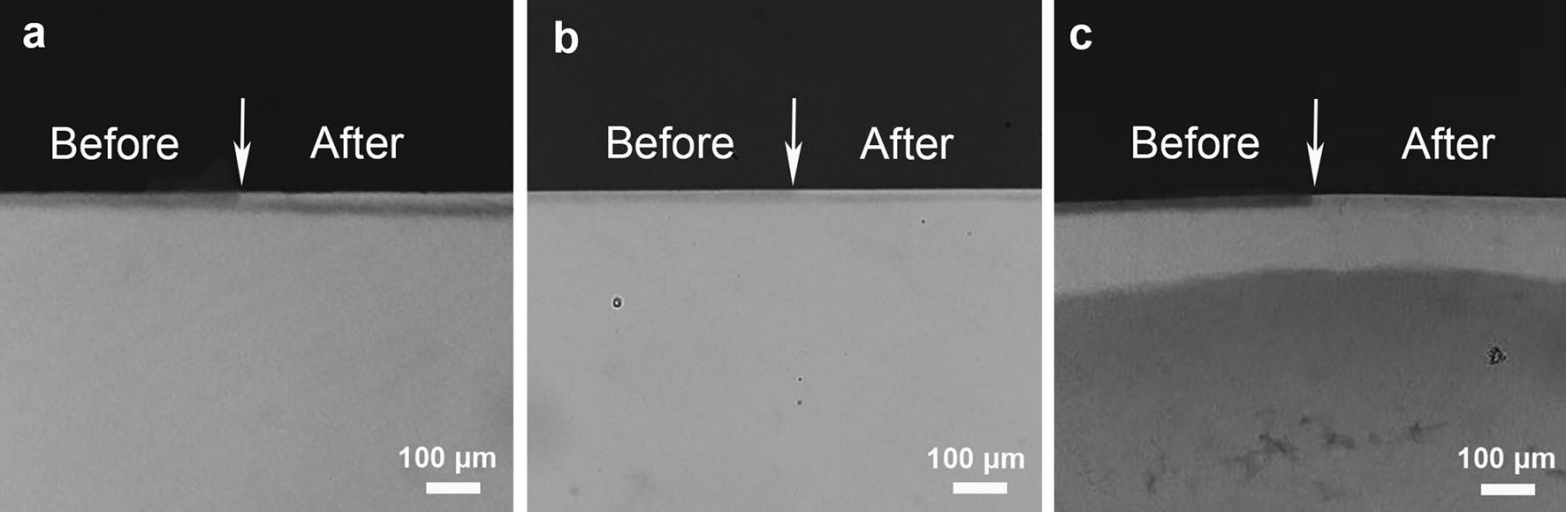
TMR microradiographs of enamel sections before and after 14 days pH cycling. (**a**) double distilled water (DDW) alone, (**b**) GERM CLEAN, (**c**) 1000 ppm fluoride (NaF). White arrows indicated the boundary before and after the pH cycling. The DDW group showed the deepest lesion depth and the lowest density of the lesion.

Representative TMR microradiographs were shown in Fig. 3. Although obvious remineralization layers could be found on the demineralized enamel surface in all groups, all the medication-treated groups showed a lower demineralization degree than that of the DDW group. The statistics data of mineral loss and lesion depth in each group were shown in Table 3. Mineral loss in the DDW group was 1342.2 [± 122.1] Vol% × µm, which was the most prominent (p = 0.001), while figures for the GC group and the NaF group were less significant, which were 921.0 [± 187.2] Vol% × µm and 489.0 [± 140.2] Vol% × µm, respectively (p = 0.001). For the lesion depth, the results of the pairwise comparison in each group were similar to those of mineral loss. The DDW group had the deepest lesion depth of 69.3 [± 21.8] µm, the GC group had a shallower lesion depth of 33.2 [± 4.3] µm than the DDW group (p = 0.049), and the NaF group had the shallowest lesion depth of 17.4 [± 3.0] µm (p = 0.001).

**Table 3:** The estimated mineral loss and lesion depth after pH cycling (Mean [± SD]).

	DDW	GC	NaF
Lesion depth (μm)	69.3 [± 21.8]^a^	33.2 [± 4.3]^b^	17.4 [± 3.0]^c^
Mineral loss (Vol%. μm)	1342.2 [± 122.1]^a^	921.0 [± 187.2]^b^	489.0 [± 140.2]^c^

Different superscript letters indicate a significant difference between groups (p < 0.05). *DDW* samples treated with double distilled water, *GC* samples treated with GERM CLEAN, *NaF* samples treated with 1000 ppm fluoride.

## Discussion

According to the results of this study, the null hypothesis was rejected. The remineralization effect of GERM CLEAN was significantly better than that of DDW. GERM CLEAN, a new biological polypeptide oral spray, occupies a certain market in China as a mature product. According to the manufacturer's instructions, the active ingredients of GERM CLEAN are oral germicide antimicrobial peptide and JN-01 antimicrobial peptide. This spray has a broad-spectrum antibacterial effect, is harmless to probiotics, and can promote mucosal repair. The antibacterial effects on *Streptococcus mutans*^22^ and dual-species biofilm formed by *Streptococcus mutans* and *Candida albicans*^23^ of this product were confirmed by previous studies. It was reported that GERM CLEAN at 1/2 minimal inhibitory concentration could reduce the acidogenicity, exopolysaccharides synthesis, adherent ability, and biofilm formation of *S. mutans* through downregulating the expression levels of gtfb, gtfc, gtfd, and ldh genes^22^. Moreover, GERM CLEAN may affect the yeast–hyphal transformation ability of *C. albicans* in dual-species biofilm which could decrease the dual-species biofilm pathogen^23^.

The remineralization effect of GERM CLEAN was investigated in the present experiment. According to the result of SMHR%, the GC group was better than the DDW group. This phenomenon indicated that GERM CLEAN could promote mineral deposition on the enamel surface to a certain extent. The results of TMR also supported this result. Studies confirmed that the mineral content was positively correlated with microhardness^27,28^, which was consistent with our results that the GC group had higher SMHR% and less mineral loss than the DDW group. However, it should be clear that SMH could be affected by the degree of mineralization and thickness of the surface layer, the extent of the subsurface (de)mineralization, and mineral distribution^28^. Actually, the organic and inorganic content of enamel from the surface to the enamel-dentinal junction is different^29^. Thus, the thickness of the enamel may also affect the result. In addition, the outcome obtained from TMR is semi-quantitative because the mineral volume measured by the commercialized software provides a proxy mineral volume, and there was some mineral loss during the grinding process. So, the results were not the actual mineral volume, only were used as a reference for determining lesion depth and mineral loss. However, studies have shown that TMR is still the gold standard for studying demineralization and remineralization of tooth hard tissue due to its relatively good accuracy and reproducibility^30^. Thus, the technique has been widely used^31–34^. For the surface roughness, GERM CLEAN also manifested a better effect than DDW, this result also confirmed the remineralization effect of GERM CLEAN.

In the present research, the observed remineralization effect of GERM CLEAN was weaker than the peptides reported in the previous studies^19,35,36^, the reason may be that GERM CLEAN has weak acidity with a pH of 6.5. The previous study reported that acidic amino acids could regulate the orientation of hydroxyapatite cyrstals^37^. The peptides with obvious mineralization effects such as QP5^19^ and 8DSS^36^ reported in the literature could promote the orientated nucleation of hydroxyapatite crystals, or could promote uniform deposition of calcium phosphate nanocrystals. The binding of peptides to hydroxyapatite is important to promote mineralization. We speculated that GERM CLEAN could bind to hydroxyapatite to limit the release of calcium and phosphate ions from the demineralized enamel while promoting the deposition of calcium and phosphate ions in the solution on the demineralized enamel to realize remineralization. However, whether it was a simple deposition of mineral ions or the formation of new hydroxyapatite still needed further investigation. According to the latest review^38^, there are forty-three synthetic antibacterial peptides, but only four antibacterial peptides promoting remineralization or preventing demineralization including Sp-H5^35^, CS-QP5^39^, TVH19^25^, and DR9-RR14^40^, and the four studies used laboratory or animal models. More clinical researches are needed before these antibacterial peptides are used for clinical treatment, while GERM CLEAN has been a clinically available antibacterial peptide.

The previous study showed that GERM CLEAN could inhibit bovine incisor enamel demineralization^23^. In our current study, GERM CLEAN could enhance the remineralization of demineralized enamel, but its mineralization effect is still unsatisfactory because its mineralization effect is significantly weaker than that of fluoride. The poor stability of GERM CLEAN according to the previous study^22^ may be another limitation and the underlying mechanisms of this phenomenon are still under investigation.

The biomimetic mineralization of enamel using peptides is still in research, and it is still far from clinical transformation. As a marketed antibacterial peptide, GERM CLEAN is a mature product, and the clinical safety of GERM CLEAN can be guaranteed. GERM CLEAN can inhibit the acid production of *Streptococcus mutans* to change the microenvironment of biofilms and prevent enamel demineralization. In addition, GERM CLEAN can also promote the remineralization of demineralized enamel. Hence, GERM CLEAN still has broad application prospects in the clinical practice of early enamel caries.

## Conclusion

Within the limitations of this study, GERM CLEAN has the potential to promote the remineralization of demineralized enamel to a certain extent, which can provide a new method for the treatment of early enamel caries.

## Data Availability

The data that support the findings of this study are available from the corresponding author upon reasonable request.
